# Factors associated with exclusive breastfeeding by maternal HIV status: a population-based survey in Kenya

**DOI:** 10.1186/s13006-024-00651-y

**Published:** 2024-06-26

**Authors:** Mame M. Diakhate, Jennifer A. Unger, Agnes Langat, Benson Singa, John Kinuthia, Janet Itindi, Edward Nyaboe, Grace C. John-Stewart, Christine J. McGrath

**Affiliations:** 1 Department of Global Health, University of Washington, 3980 15th Ave NE, Box 351620, Seattle, WA 98195 USA; 2 Department of Obstetrics and Gynecology, Women and Infants Hospital, Brown University, Providence, RI USA; 3 Centers for Disease Control and Prevention Kenya, Nairobi, Kenya; 4 Kenya Medical Research Institute, Nairobi, Kenya; 5 Departments of Obstetrics and Gynaecology, Research & Programs, Kenyatta National Hospital, Nairobi, Kenya; 6 Department of Epidemiology, University of Washington, Seattle, WA USA; 7 Department of Medicine, University of Washington, Seattle, WA USA; 8 Department of Pediatrics, University of Washington, Seattle, WA USA

**Keywords:** Exclusive breastfeeding, Women living with HIV, HIV-exposed uninfected children, Immunization visits, Breastfeeding, Sub-saharan Africa

## Abstract

**Background:**

Exclusive breastfeeding (EBF) in the first six months remains low globally, despite known benefits of lower morbidity and mortality among breastfed infants. It is important to understand factors associated with breastfeeding to support optimal breastfeeding practices, particularly in settings with a high burden of HIV.

**Methods:**

We analyzed data from a population-level survey of mother-infant pairs attending 6-week or 9-month immunizations at 141 clinics across Kenya. Primary outcomes included maternal report of (1) EBF at 6-week visit, defined as currently feeding the infant breast milk only, (2) EBF for the first 6-months of life, defined as breastfeeding or feeding the infant breast milk only with no introduction of other liquids or solid foods until 6 months, and (3) continued breastfeeding with complementary feeding at 9-months. Correlates of breastfeeding practices were assessed using generalized Poisson regression models accounting for facility-level clustering.

**Results:**

Among 1662 mothers at 6-weeks, nearly all self-reported breastfeeding of whom 93% were EBF. Among 1180 mothers at 9-months, 99% had ever breastfed, 94% were currently breastfeeding and 73% reported 6-month EBF. At 6-weeks, younger age (< 25 years) (adjusted Prevalence Ratio (aPR) 0.96; 95% CI 0.93, 0.99), lower education (aPR 0.96; 95% CI 0.93, 0.99) and recent infant illness (aPR 0.97; 95% CI 0.94, 1.00) were associated with lower EBF prevalence while women living with HIV (WLWH) had higher EBF prevalence (aPR 1.06; 95% CI 1.02, 1.10) than women without HIV. 6-month EBF prevalence was 26% higher in WLWH (aPR 1.26; 95% CI 1.15, 1.35) than women without HIV, 14% lower in women reporting mild or above depressive symptoms (aPR 0.86; 95% CI 0.76, 0.99) than those with none or minimal depressive symptoms, and 15% lower in women with versus without history of intimate partner violence (aPR 0.85; 95% CI 0.74, 0.98). At 9-months, WLWH had a lower prevalence of continued breastfeeding with complementary feeding (aPR 0.73; 95% CI 0.64, 0.84) than women without HIV.

**Conclusion:**

WLWH had higher EBF prevalence in the first 6-months, but lower prevalence of continued breastfeeding at 9-months. Strategies to support EBF and continued breastfeeding beyond 6-months postpartum, particularly among WLWH, are needed.

**Supplementary Information:**

The online version contains supplementary material available at 10.1186/s13006-024-00651-y.

## Background

Breastfeeding is the optimal infant feeding practice. Breast milk contains essential nutrients, anti-inflammatory components, and prebiotics to support infant health, growth, and cognitive development [1]. Exclusive breastfeeding (EBF) has been shown to reduce diarrheal illness, pneumonia, and death in early childhood as well as reduce the risk of obesity in later childhood [2–5]. The World Health Organization (WHO) recommends EBF for the first 6 months of life and continued breastfeeding with nutritionally adequate and safe complimentary feeds for 24 months or beyond [6]. Despite these recommendations, less than 40% of infants globally are EBF for 6 months and only 73% of infants are receiving any breast milk at one year of age [1]. According to the Kenya Demographic and Health Survey, the prevalence of EBF among infants less than 6 months was 61% in 2014 and 60% in 2022 [7].

Factors that influence a woman’s decision to breastfeed are complex and multifaceted. In high income countries, higher socioeconomic status and education are associated with breastfeeding initiation and duration [1]. In contrast, in low- and middle-income countries (LMICs), mothers with lower income are more likely to breastfeed for longer periods of time than wealthier mothers [1]. These practices are further influenced by cultural beliefs and community norms. A study in Bangladesh reported higher prevalence of EBF in women identifying as a housewife [8]. A recent systematic review largely from high income countries found that younger age and lower education levels were associated with cessation of EBF before 6 months postpartum and returning to work within 12-weeks of delivery was associated with cessation of breastfeeding before 6 months postpartum [9]. Partner support has also been shown to increase adherence to EBF, particularly in LMIC [10].

Psychosocial and health-related factors influence breastfeeding practices. Marital and financial-related stress and relocating during pregnancy have been associated with early breastfeeding cessation [11]. Mothers in Barbados reporting postpartum depressive symptoms were less likely to believe that breastfeeding was better for their infants [12]. A woman’s HIV status may further influence her decision to breastfeed.

Despite the known benefits and WHO breastfeeding recommendations for women living with HIV (WLWH) which include EBF for the first 6 months and continued breastfeeding up to 2 years and beyond with maternal and infant receipt of antiretroviral interventions [13], data on uptake and duration of EBF are mixed. A study in eastern Kenya reported that WLWH were less likely to EBF for 6 months if it was not the norm in their community or if the child was also being cared for by other family members [14]. In addition, stigma and fear of being suspected of having HIV has been shown to influence a mother’s decision to not exclusively breastfeed [14]. WLWH may also be at higher risk of depression, and depression has been associated with lower rates of EBF in LMICs [15, 16]. However, a study in western Kenya, an area with higher HIV prevalence, found that WLWH were more likely to EBF for the first 6 months than women who were not living with HIV [17].

Limited population-level data are available on the prevalence and determinants of breastfeeding practices among women in LMIC, particularly in settings with a high burden of HIV. In areas with high immunization uptake, such as Kenya, infant immunization programmes provide a unique opportunity to assess and support breastfeeding women and their families. We examined and compared factors associated with breastfeeding practices in women with and without HIV attending week 6 or month 9 infant immunizations in Kenya.

## Methods

### Study design and population

We used data from two facility-based cross-sectional surveys evaluating prevention of mother-to-child transmission of HIV and maternal child health (PMTCT MCH 2013) programs at 141 facilities in Kenya from July to December 2013 (Additional Fig. 1) [18]. The primary survey used probability proportionate to size sampling to randomly select 120 facilities across Kenya. A second survey enrolled only WLWH and their infants at 30 randomly selected facilities in Nyanza province, an area of high HIV prevalence, as previously described [18]. Mother-infant pairs attending week 6 or month 9 immunizations were eligible for participation.

Detailed study methods have been described elsewhere [18]. Briefly, the national survey consecutively enrolled all consenting mother-infant pairs attending week 6 or month 9 immunizations during a preset 5-day period, regardless of maternal HIV status. The Nyanza survey consecutively enrolled all consenting WLWH and their infants at week 6 or month 9 immunizations during a 10-day period. If a facility was included in both the national and Nyanza survey, mother-infant pairs were enrolled during a consecutive 15-day period and not eligible to re-enroll. Infants brought to the facility by someone other than their biological mother and mothers of infants not receiving immunizations at week 6 or month 9 were not eligible for enrollment in the study.

### Data collection

Trained mobile study teams administered standardized questionnaires that included questions on maternal and paternal demographics, household characteristics, antenatal care, reproductive and family planning history, maternal HIV status and use of antiretroviral therapy (ART) or antiretroviral drugs (ARVs) among WLWH, birth outcomes, breastfeeding, and infant HIV testing. Maternal and infant anthropometry were measured by study staff. Responses to the Patient Health Questionnaire-9 (PHQ-9) depression scale [19, 20] were used to categorize maternal depression symptoms as “mild, moderate or severe” (combined scores ≥ 5) and “minimal or none” (scores < 5). The Hurt, Insult, Threaten, and Scream (HITS) intimate partner violence (IPV) tool [21] was used to assess history of IPV. Women who scored ≥ 10 on the HITS were categorized as experiencing or having experienced IPV. HIV stigma was defined as experiencing any perceived internal and or external stigma and discrimination based on a previously validated HIV stigma survey from Africa [22]. Maternal illness was defined as report of fever, cough, weight loss, failing to gain weight, night sweats, fatigue, loss of appetite or coughing blood in the past month. Breast health problems were defined as presence of mastitis, abscess, swelling, nipple bleeding, or nipple cracking since the birth of the child. HIV results among mothers self-reporting HIV-positive status were verified by the Maternal and Child Health booklet. Women of unknown or negative HIV status were offered rapid HIV antibody testing using Determine Test Kit® (Abbott Laboratories, Chicago, Illinois). Women whose HIV status was unknown and mother-infant pairs in which the child tested HIV positive were excluded from the analysis. Ten women (< 1% of enrollees) who reported their child was never breastfed or fed any breast milk were excluded from the analysis. Of these ten women, two were women without HIV (attending month 9 immunizations) and eight were WLWH (six at week 6 and two at month 9 immunizations). Of the eight WLWH who never breastfed, seven reported being on ART and one reported not being on ART.

### Ethical considerations

The study was approved by ethical review boards at the University of Washington (UW IRB No. 41,953) and Kenya Medical Research Institute (KEMRI ERC No. 2200). The study was reviewed according to the US Centers for Disease Control and Prevention (CDC) human research protection procedures and was determined to be research, but CDC was not engaged. Informed consent was obtained from all participants prior to enrollment.

### Breastfeeding outcomes

EBF at the 6-week immunization visit was categorized as “yes” if the mother responded “yes” to “Are you currently breastfeeding your infant?” and “breastfeeding or breast milk only” when asked “How would you describe your current infant feeding practice?”. If the mother responded to currently feeding their child breast milk and water, tea, juice, formula or other non-human milk, or solids this was categorized as not EBF at the 6-week visit.

At the 9-month visit, currently breastfeeding was categorized as “yes” if the mother responded “yes” to “Are you currently breastfeeding your infant?”. If the mother responded “yes” to “Did you ever breastfeed your child” and when asked “At what age did the child first start receiving any other liquid or solid foods?” responded that the infant was at least 6 months of age, this was categorized as EBF for the first 6 months. If the mother responded that the infant stopped breastfeeding or was introduced other liquids or solids before 6 months of age, this was categorized as not having EBF for the first 6 months.

### Statistical analysis

All women with known HIV status at the 6-week or 9-month immunization visit were included in the analysis. Given the sampling design and to account for variation in the estimates, means or proportions and 95% confidence intervals (CI) are presented for continuous and categorical variables, respectively. Poisson generalized linear models with log-link function were used to determine the prevalence ratios (PR) and 95% CI. Covariates associated with breastfeeding outcomes at p-value < 0.10 in univariate analysis were included in multivariate models. A p-value < 0.05 was considered statistically significant. In secondary analysis, we evaluated factors that were associated with breastfeeding practices stratified by maternal HIV status. All analyses accounted for clustering at the facility level and were conducted using Stata 16 (StataCorp LLC, College Station, Texas, USA).

## Results

### Maternal characteristics

Of 2,891 mother-infant pairs surveyed, 49 were excluded due to unknown maternal HIV status. Hence, 2,842 were included in this analysis. Of these, 1,662 (58%) were attending 6-week immunizations and 1,180 (42%) were attending 9-month immunizations. Mean maternal age was 26 years (95% CI 25.4, 26.7) with no difference in mean age by visit (Table 1). Most (87%) mothers were married and about 55% had less than secondary education. About 90% of all mothers reported that their partner or the child’s father provided financial support to the mother and her infant. 11% of mothers at 6-weeks and 13% of mothers at 9-months reported experiencing mild, moderate or severe depressive symptoms. 16% of mothers at 6-weeks and 20% of mothers at 9-months were living with HIV (Table 1).

**Table 1:** Maternal and infant characteristics at 6-week and 9-month immunization visits^a^

	% or Mean (95% Confidence Interval)	
	6-week (*n* = 1662)	9-month (*n* = 1180)
**Maternal characteristics**		
Age (years)	25.7 (25.4, 26.0)	26.3 (25.9, 26.7)
<25 years	47.0% (44.4, 49.8)	43.8% (40.6, 47.1)
Women living with HIV	15.6% (12.1, 20.0)	20.2% (15.2, 26.2)
Married or cohabiting	86.6% (84.7, 88.4)	86.5% (84.3, 88.5)
Primary education and below	55.4% (51.5, 59.1)	55.9% (51.4, 60.2)
Employment		
Salaried	10.0% (8.4, 11.9)	11.4% (9.4, 13.7)
Self-employed	26.9% (24.3, 29.6)	28.7% (25.8, 31.8)
Unemployed/housewife	63.1% (60.0, 66.1)	59.9% (56.6, 63.2)
Partner provides financial support	91.2% (89.7, 92.4)	89.7% (87.7, 91.3)
Depressive symptoms: Mild, moderate or severe	10.5% (8.3, 13.2)	13.1% (10.6, 16.3)
Intimate partner violence (HITS score ≥ 10)	8.4% (6.7, 10.6)	11.1% (9.0, 13.6)
Maternal illness in past month ^b^	30.1% (26.0, 34.7)	36.0% (31.3, 41.0)
Crowding (3 + persons per room)	41.5% (37.0, 46.1)	43.5% (39.6, 47.5)
Number of children	3.0 (2.9, 3.1)	3.0 (2.9, 3.2)
One child	7.0% (5.4, 9.0)	7.4% (5.7, 9.5)
Two or more children	93.0% (91.0, 94.6)	92.6% (90.5, 94.3)
Underweight (BMI < 18.5)	4.9% (3.5, 6.7)	5.4% (4.1, 7.2)
HIV Stigma (among all women)		
Internal stigma	29.9% (26.4, 33.6)	31.8% (27.7, 36.2)
External stigma	57.7% (52.2, 63.0)	59.4% (53.3, 65.2)
**Breastfeeding characteristics**		
Currently breastfeeding	99.7% (99.3, 99.9)	93.5% (91.2, 95.2)
Exclusive breastfeeding	92.5% (90.4, 94.2)	---
Exclusive breastfeeding for 6 months	---	73.2% (69.7, 76.5)
Initiated breastfeeding < 24 h of birth	95.7% (94.2, 96.9)	94.6% (92.4, 96.3)
Infant age (months) introduced other foods/liquids	1.1 (0.9, 1.3)	5.4 (5.3, 5.5)
Breast health problems ^c^	9.4% (7.9, 11.1)	10.2% (8.2, 12.5)
**Infant characteristics**		
Low birth weight (< 2.5 kg)	6.0% (4.9, 7.2)	5.3% (4.0, 7.0)
Prior hospitalization	4.2% (3.0, 5.6)	11.5% (9.2, 14.4)
Illness in past month	42.4% (37.9, 47.1)	63.8% (58.3, 69.0)
Difficulty breathing	12.8% (10.3, 15.6)	13.7% (11.4, 16.5)
Difficulty feeding	2.2% (1.6, 3.1)	14.0% (11.1, 17.5)
Fever	14.6% (11.9, 17.7)	36.5% (32.0, 41.3)
Poor appetite	1.5% (1.0, 2.3)	19.3% (16.1, 23.0)
Diarrhea	5.1% (3.8, 6.7)	19.7% (16.6, 23.1)

*Abbreviations* CI, confidence interval

^a^ Analyses account for sample design and facility level clustering and include only mothers who ever breastfed (10 mothers reporting that they did not initiate breastfeeding were excluded due to small sample size); ^b^ Maternal illness includes fever, prolonged fever, cough, prolonged cough, weight loss, failing to gain weight, night sweats, fatigue, loss of appetite and coughing blood; ^c^ Presence of any of the following: mastitis, abscess, swelling, nipple bleeding, nipple cracking

### Breastfeeding characteristics

Of the mother-infant pairs attending 6-week immunizations and who had initiated breastfeeding, 100% of women reported currently breastfeeding and about 93% reported EBF (Table 1). Nearly all (96% of WLWH and 92% of women without HIV) reported EBF among this 6-week immunization group (Additional Table 1). About 96% of the women reported initiating breastfeeding within 24 h of birth. Among those not EBF, the mean infant age at introduction to other feeds was about one month (4.8 weeks; 95% CI 3.9, 5.6) (Table 1).

Among mothers at the 9-month infant immunization visit, 94% (95% CI 91.2, 95.2) reported continued breastfeeding with complementary feeds. Mean infant age at introduction to other foods was 5.3 months (95% CI 5.2, 5.4) for women not living with HIV and 5.7 months (95% CI 5.5, 5.9) for WLWH (*p* = 0.05) (Additional Table 1). Most (95%) women reported initiating breastfeeding within 24 h of birth (Table 1).

Nearly three-quarters (73%; 95% CI 69.7, 76.5) of women at the 9-month visit reported EBF their infants during the first 6 months of life. A significantly higher prevalence (86%; 95% CI 80.4, 90.4) of WLWH reported 6-month EBF than women not living with HIV (70%; 95% CI 66.2, 73.5; *p* < 0.001) (Additional Table 1).

### Factors associated with breastfeeding practices

In univariate analyses, among mothers at the 6-week infant immunization, younger age (PR 0.96; 95% CI 0.93, 0.99, *p* = 0.006), lower educational attainment (PR 0.96; 95% CI 0.93, 0.99, *p* = 0.003), history of IPV (PR 0.95; 95% CI 0.89, 1.00, *p* = 0.068), and maternal and infant illness in the past month (PR 0.96, 95% CI 0.92, 1.00, *p* = 0.041 and PR 0.95; 95% CI 0.92, 0.99, *P* = 0.006, respectively) were associated with lower EBF prevalence (Table 2). WLWH had higher prevalence of EBF at 6-weeks than women not living with HIV (PR 1.05; 95% CI 1.02, 1.09, *p* = 0.004). In multivariate model, younger age, lower educational attainment, recent infant illness, and maternal HIV status remained significantly associated with EBF prevalence at 6-weeks (Table 3).

**Table 2:** Univariate analysis of associations between factors and breastfeeding practices at 6-week and 9-month immunization visits ^a^

	Exclusive breastfeeding at 6-week visit ^b^		Exclusive breastfeeding for 6-months ^c^		Continued breastfeeding at 9-month visit ^d^	
	PR (95% CI)	*P*-value	PR (95% CI)	*P*-value	PR (95% CI)	*P*-value
Age < 25 years (ref: ≥25)	0.96 (0.93, 0.99)	0.006	0.97 (0.91, 1.04)	0.453	1.06 (1.02, 1.10)	0.001
Women living with HIV (ref: HIV-negative)	1.05 (1.02, 1.09)	0.004	1.23 (1.14, 1.32)	< 0.001	0.74 (0.66, 0.84)	< 0.001
BMI (kg/m^2^) < 18.5 (ref: ≥18.5)	0.97 (0.91, 1.05)	0.452	0.98 (0.84, 1.15)	0.809	0.99 (0.92, 1.06)	0.682
One child (ref: ≥2)	1.00 (0.93, 1.06)	0.919	1.04 (0.87, 1.24)	0.683	1.05 (0.95, 1.16)	0.359
Maternal illness in past month	0.96 (0.92, 1.00)	0.041	0.99 (0.91, 1.08)	0.891	1.00 (0.96, 1.04)	0.952
Breast problems (ref: none)	0.95 (0.90, 1.01)	0.104	0.88 (0.76, 1.01)	0.077	1.03 (0.99, 1.07)	0.197
Primary education and below (ref: above primary)	0.96 (0.93, 0.99)	0.003	0.90 (0.83, 0.97)	0.007	1.00 (0.96, 1.03)	0.849
Married/cohabiting (ref: other)	1.02 (0.98, 1.06)	0.305	0.97 (0.89, 1.07)	0.594	1.06 (1.00, 1.13)	0.034
Mild, moderate or severe depression (ref: minimal or none)	0.98 (0.93, 1.03)	0.429	0.87 (0.76, 0.99)	0.032	0.95 (0.91, 1.00)	0.063
Intimate partner violence HITS score ≥ 10 (ref: <10)	0.95 (0.89, 1.00)	0.068	0.86 (0.75, 0.99)	0.037	0.98 (0.93, 1.03)	0.411
Partner provides financial support (ref: no)	1.03 (0.98, 1.08)	0.302	1.04 (0.93, 1.17)	0.460	1.05 (0.99, 1.12)	0.096
Low birth weight (ref: ≥2.5 kg)	0.93 (0.85, 1.02)	0.116	0.99 (0.83, 1.18)	0.881	0.90 (0.80, 1.01)	0.082
Prior infant hospitalization	0.97 (0.89, 1.05)	0.449	1.00 (0.89, 1.13)	0.938	0.98 (0.93, 1.04)	0.490
Infant illness, past mo.	0.95 (0.92, 0.99)	0.006	0.88 (0.81, 0.95)	0.002	1.05 (1.01, 1.09)	0.012

*Abbreviations* PR, prevalence ratio; CI, confidence interval

^a^ Analyses account for sample design and facility level clustering and include only mothers who ever breastfeeding (10 women reporting that they did not initiate breastfeeding were excluded)

^b^ Exclusive breastfeeding at 6-week visit included only mothers attending 6-week immunizations

^c^ 6-month exclusive breastfeeding was asked at the month-9 immunization visit

^d^ Continued breastfeeding at 9-month visit included only mothers attending 9-month immunizations

**Table 3:** Multivariable analysis of factors associated with breastfeeding practices at 6-week and 9-month infant immunizations ^a^

	Exclusive breastfeeding at 6-week visit^b^		Exclusive breastfeeding for 6-months^c^		Continued breastfeeding at 9-month visit^d^	
	Adjusted Prevalence Ratio (95% CI)	*P*-value	Adjusted Prevalence Ratio (95% CI)	*P*-value	Adjusted Prevalence Ratio (95% CI)	*P*-value
Maternal age < 25 years (ref: ≥25 years)	0.96 (0.93, 0.99)	0.013			1.02 (0.99, 1.06)	0.188
Women living with HIV (ref: HIV-negative)	1.06 (1.02, 1.10)	0.002	1.26 (1.15, 1.35)	< 0.001	0.73 (0.64, 0.84)	< 0.001
Maternal illness in past month (re: none)	0.97 (0.93, 1.01)	0.116				
Breast problems (ref: none)			0.91 (0.79, 1.05)	0.199		
Primary education and below (ref: above primary)	0.96 (0.93, 0.99)	0.006	0.89 (0.82, 0.96)	0.002		
Married/cohabiting (ref: other)					1.06 (1.00, 1.12)	0.059
Mild, moderate or severe depressive symptoms (ref: minimal or none)			0.86 (0.76, 0.99)	0.030	0.98 (0.92, 1.03)	0.389
Intimate partner violence ≥ 10 HITS score (ref:<10)	0.95 (0.89, 1.02)	0.158	0.85 (0.74, 0.98)	0.024		
Low birth weight (ref: ≥2.5 kg)					0.91 (0.82, 1.00)	0.044
Infant illness in past month (ref: none)	0.97 (0.94, 1.00)	0.034	0.92 (0.85, 0.99)	0.037	1.04 (1.00, 1.08)	0.054

^a^ Analyses account for sample design and facility level clustering. Multivariable model included factors univariately associated with breastfeeding practices at *p* < 0.10; only mothers reporting ever breastfeeding were included in the analyses (10 women who did not initiate breastfeeding were excluded)

^b^ Exclusive breastfeeding at 6-week visit included only mothers at 6-week immunizations: age, HIV status, maternal illness in past month, education, intimate partner violence, infant illness

^c^ 6-month exclusive breastfeeding was asked at the month-9 immunization visit: HIV status, breast problems, education, depressive symptoms, intimate partner violence, infant illness

^d^ Continued BF at 9-month visit included only mothers at 9-month immunizations: age, HIV status, marital status, depressive symptoms, low birth weight, infant illness

Among those attending the 9-month immunization, maternal HIV status (PR 0.74, 95% CI: 0.66, 0.84; *p* < 0.001) was associated with lower prevalence of continued breastfeeding while recent infant illness (PR 1.05, 95% CI: 1.01, 1.09; *p* = 0.012) was associated with a higher prevalence of continued breastfeeding (Table 2). In the multivariate analysis, maternal HIV status, low birth weight, marital status, and infant illness remained significant (Table 3). Maternal age and depressive symptoms were no longer significant in adjusted analysis.

Lower prevalence of 6-month EBF was associated with lower educational attainment (PR 0.90, 95%CI: 0.83, 0.97, *p* = 0.007), mild, moderate or severe depression (PR 0.87, 95% CI: 0.76, 0.99; *p* = 0.032), history of IPV (PR 0.86, 95% CI: 0.75, 0.99; *p* = 0.037), and recent infant illness (PR 0.88, 95% CI: 0.81, 0.95; *p* = 0.002) in univariate and adjusted analyses. WLWH had a higher 6-month EBF prevalence (PR 1.23; 95% CI 1.14, 1.32; *p* < 0.001) than women not living with HIV (Table 2). This association remained after adjusting for breast problems, education, depressive symptoms, history of IPV, and infant illness (see Table 3).

As maternal HIV status was associated with both higher and lower prevalence of breastfeeding behaviors at different time point, we further evaluated factors associated with optimal breastfeeding practices stratified by HIV status.

### Factors associated with breastfeeding practices among women with HIV

Among WLWH at 6-week infant immunizations, younger age (PR 0.92; 95% CI 0.84, 1.00; *p* = 0.044), maternal BMI of < 18.5 kg/m^2^ (PR 1.04; 95% CI 1.01, 1.07; *p* = 0.006), having only one child (PR 0.97; 95% CI 0.94, 1.00; *p* = 0.03), and prior infant hospitalization (PR 1.04; 95% CI 1.01, 1.07; *p* = 0.006) were associated with EBF. In multivariate analysis, maternal age, BMI and number of children remained statistically significant (Additional Table 2).

Among WLWH attending 9-month infant immunizations a history of IPV (PR 1.16; 95% CI 0.99, 1.35; *p* = 0.063), infant illness in the past month (PR 1.19; 95% CI 0.97, 1.44; *p* = 0.088) and low birth weight (PR 0.50; 95% CI 0.25, 0.99; *p* = 0.048) were associated with continued breastfeeding. Both, a history of IPV (aPR 1.18; 95% CI 0.98, 1.42; *p* = 0.083), and low birth weight (aPR 0.50; 95% CI 0.25, 0.99; *p* = 0.046) remained statistically significant in the multivariate analysis.

WLWH experiencing mild, moderate or severe depressive symptoms at 9-months postpartum reported a lower prevalence of 6-month EBF than WLWH reporting no depression (PR 0.79; 95% CI 0.65, 0.97; *p* = 0.024) (Additional Table 2). However, no other factors were found to be statistically significant with 6-month EBF.

### Factors associated with breastfeeding practices among women living without HIV

Among women not living with HIV at 6-weeks, lower education levels (PR 0.95; 95% CI 0.92, 0.98; *p* = 0.004), maternal illness (PR 0.94; 95% CI 0.89, 0.99; *p* = 0.016), infant illness (PR 0.95; 95% CI 0.91, 0.99; *p* = 0.007), maternal age (PR 0.97; 95% CI 0.94, 1.00; *p* = 0.062) and a history of IPV (PR 0.93; 95% CI 0.86, 1.01; *p* = 0.07) were associated with EBF. All associations, except IPV, remained significant in the multivariate analysis (Additional Table 3).

Among women not living with HIV, 6-month EBF prevalence was lower in those with lower education levels (aPR 0.87; 95% CI 0.79, 0.95; *p* = 0.002), history of IPV (aPR 0.78; 95% CI 0.64, 0.95; *p* = 0.015), and recent infant illness (aPR 0.89; 95% CI 0.80, 0.98; *p* = 0.016). (Additional Table 3).

Among women not living with HIV at the month 9 visit, no significant associations were found between maternal factors and continued breastfeeding. (Additional Table 3).

## Discussion

In this cohort of 2,842 women from two different surveys across Kenya, the prevalence of EBF at 6-weeks and continued breastfeeding at 9-months was 93% and 94%, respectively. WLWH had 25% higher prevalence of EBF during the first 6-months of life than women not living with HIV. However, at 9-months, the prevalence of continued breastfeeding was 27% lower in WLWH compared to their HIV-uninfected counterparts. Lower educational attainment was associated with lower prevalence of EBF at 6-weeks and 6-month EBF. Mild, moderate or severe depressive symptoms and history of IPV were associated with lower prevalence of continued EBF at 6 months.

Among WLWH, increased coverage of antiretroviral therapy and changing perceptions on HIV may have led to changes in behavior in breastfeeding uptake and continuation. Our study demonstrates the success of this effort with over 90% of WLWH reporting EBF at 6-weeks and 86% reporting EBF for 6-months. A breastfeeding peer counseling study among WLWH in Kenya reported EBF prevalence of 83% at 14 weeks [23]. Whereas, in the general Kenya population, exclusive breastfeeding in children under age 6 months, reported as having been exclusively fed with breast milk in the prior day, increased from 32% in 2008–2009 to 61% in 2014 and remained at 60% in 2022 [7, 24]. Interestingly, our results show a higher EBF prevalence than those reported in studies conducted in a similar period among WLWH in Kilifi County, Kenya (50% exclusively breastfeeding) and Western Kenya [14, 17]. Our study included WLWH attending immunization facilities across Kenya, including high HIV prevalence counties. The high prevalence of EBF we observed may be a result of successful healthcare worker training and reinforced messaging and educational awareness of 6-months EBF among WLWH. Alternatively, this reinforced messaging may have led WLWH to self-report that they exclusively breastfed for 6-months, even if they had not, due to social desirability or recall bias.

Although our data demonstrate good EBF practices among WLWH, we found a lower prevalence of continued breastfeeding among WLWH at 9-months postpartum compared to their HIV-uninfected peers. Between 2000 and 2013, there were several changes to breastfeeding guidelines for WLWH. It is possible that this led to inconsistent messaging from healthcare workers related to continued breastfeeding with introduction to other feeds after 6-months of age and confusion for WLWH, particularly those women who may have had an infant during a prior policy in which continued breastfeeding beyond 6-months of age was not recommended. For instance, the 2006 WHO Infant Feeding Guidelines recommended that WLWH exclusively breastfeed their infants for the first 6 months of life followed by complementary foods and continuation of breastfeeding after 6 months if replacement feeding was not acceptable, feasible, affordable, sustainable and safe [13]. Changing guidelines has been shown to cause confusion among healthcare workers and delayed facility-level roll-out of new guidelines in rural communities [14]. Moreover, communities often hold onto norms well beyond guideline changes. Both facility-level factors and community norms could impact the type of breastfeeding information women received.

Similar to prior studies, we observed that lower education was associated with lower prevalence of EBF. Higher education was associated with better uptake of EBF to six months among women in Mali [25]. Higher educational attainment may play a role in understanding the importance of EBF during the first 6 months of life, particularly among women in communities where EBF is not the norm. Designing breastfeeding educational interventions that include women attending antenatal or postnatal care and in the community is critical to increasing uptake and duration of EBF.

We found that women reporting mild, moderate or severe depressive symptoms had lower prevalence of 6-month EBF. This is consistent with previous studies. Silva et al. reported a 1.63-fold higher chance of EBF interruption in mothers experiencing postpartum depression in Brazil [16]. Understanding how depression may impact a mother’s choice and ability to breastfeed is important for improving breastfeeding uptake and designing strategies to support these women.

Our study also found an association between infant illness and lower prevalence of EBF. This finding is similar to a study which showed that infant illness was a major reason for mothers discontinuing breastfeeding before they desired [26]. Importantly, breastfeeding has been shown to protect children from illnesses like diarrhoea and hospitalization [1].

Our study has strengths and limitations. While this study was conducted in 2013, these findings continue to be relevant as infant feeding guidelines have not substantially changed since these data were collected [27, 28]. Mother-infant pairs were enrolled in 141 facilities across Kenya, allowing for a comprehensive understanding of breastfeeding practices in urban and rural settings. Outcomes are limited by being self-reported measures. Finally, this is a facility-based survey and mothers receiving facility-based care may differ from mothers in the community. However, uptake of infant immunizations at 6-weeks and 9-months of age is high (> 90%) in Kenya [29].

## Conclusions

In conclusion, we found breastfeeding uptake and prevalence of EBF was high among women in Kenya. Maternal age, education, depressive symptoms, history of IPV, and HIV status were associated with a mother’s likelihood of breastfeeding. Strategies to support breastfeeding throughout the postpartum period including infant immunizations and HIV care visits may be needed. Screening for IPV and depression during immunization visits may also be beneficial for the uptake and practice of recommended breastfeeding. Our results underscore the importance of the promotion of appropriate breastfeeding practices and tailored strategies to support younger mothers and those at risk for depression and IPV.

### Electronic supplementary material

Below is the link to the electronic supplementary material.

Supplementary Material 1

## Data Availability

Data described in the manuscript, code book, and analytic code will be made available upon request pending application and approval.

## Abbreviations

aPR: Adjusted Prevalence Ratio
ART: Antiretroviral therapy
ARV: Antiretroviral drug
CDC: US Centers for Disease Control and Prevention
CI: Confidence interval
EBF: Exclusive breastfeeding
HITS IPV: The Hurt, Insult, Threaten, and Scream intimate partner violence tool
IPV: intimate partner violence
LMICs: low- and middle-income countries
PHQ-9: Patient Health Questionnaire-9
PR: Prevalence ratio
WHO: World Health Organization
WLWH: Women living with HIV
