# Supplementary material for: Factors associated with exclusive breastfeeding by maternal HIV status: a population-based survey in Kenya
Source: Int Breastfeed J. 2024 Jun 26;19:44. doi: 10.1186/s13006-024-00651-y (PMC11210159; doi:10.1186/s13006-024-00651-y)
Supplement: Supplementary file 1 — Supplementary Material 1 [file 13006_2024_651_MOESM1_ESM.docx]

Additional Figure 1. Distribution of survey facilities across regions of Kenya ^a^


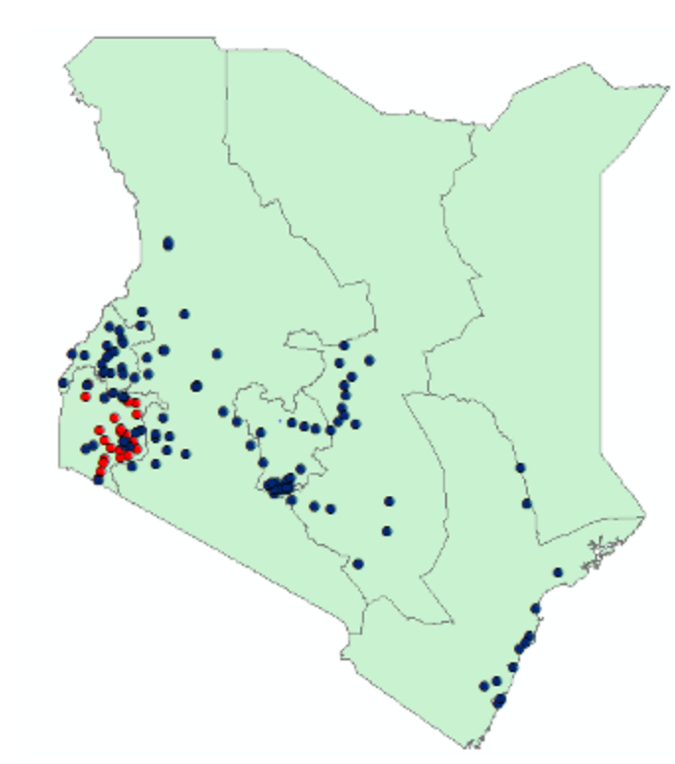


^a^ Black dots indicate facilities included in the primary survey. Red dots indicate facilities included in the Nyanza survey.

Additional Table 1. Breastfeeding characteristics at 6-week and 9-month immunization visits by HIV status^a^

| **Breastfeeding characteristics** | **% (95% CI) or Mean (95% CI)** | | | |
| --- | --- | --- | --- | --- |
|  | **6-week (n=1662)** | | **9-month (n=1180)** | |
|  | **Women not living**  **with HIV**  **(n=1402)** | **Women living**  **with HIV**  **(n=260)** | **Women not living**  **with HIV**  **(n=942)** | **Women living**  **with HIV**  **(n=238)** |
| Currently breastfeeding | 99.9% (99.5-100) | 98.4% (95.9-99.4) | 98.5% (97.5-99.1) | 73.2% (63.5-80.9) |
| Exclusive breastfeeding | 91.8% (89.4-93.7) | 96.4% (92.9-98.2) | --- | --- |
| Exclusive breastfeeding for 6 months | --- | --- | 70.0% (66.2-73.5) | 86.1% (80.4-90.4) |
| Initiated breastfeeding <24 hours of birth | 95.2% (93.4-96.5) | 98.8% (96.4-99.6) | 93.5% (90.9-95.4) | 99.2% (96.6-99.8) |
| Infant age (months) introduced other foods/liquids | 1.12 (0.9-1.4) | 1.1 (0.5-1.7) | 5.3 (5.2-5.4) | 5.7 (5.5-5.9) |
| Breast health problems ^c^ | 9.8% (8.2-11.7) | 6.9% (4.5-10.5) | 11.3% (9.1-13.8) | 5.9% (3.1-10.8) |

Abbreviations: CI, confidence interval

^a^ Analyses account for sample design and facility level clustering and include only mothers that ever breastfed (10 mothers reporting that they did not initiate breastfeeding were excluded)

Additional Table 2. Univariate and multivariable analysis of factors associated with breastfeeding practices in women living with HIV ^a^

|  | **Exclusive breastfeeding at 6-week visit^b^** | | | | **Exclusive breastfeeding during first 6-months^c^** | | **Continued breastfeeding at 9-month visit^d^** | | | |
| --- | --- | --- | --- | --- | --- | --- | --- | --- | --- | --- |
|  | **PR (95% CI)** | **P** | **aPR (95% CI)** | **aP** | **PR (95% CI)** | **P** | **PR (95% CI)** | **P** | **aPR (95% CI)** | **aP** |
| Maternal age <25 years  (ref: ≥25) | 0.92 (0.84, 1.00) | 0.044 | 0.90 (0.80, 1.01) | 0.065 | 0.96 (0.86, 1.09) | 0.545 | 1.14 (0.98-1.34) | 0.096 |  |  |
| Maternal BMI<18.5 (ref: ≥18.5) | 1.04 (1.01, 1.07) | 0.006 | 1.03 (1.00, 1.07) | 0.077 | 0.89 (0.64, 1.23) | 0.467 | 0.94 (0.68, 1.31) | 0.726 |  |  |
| One child (ref: ≥2) | 0.97 (0.94, 1.00) | 0.031 | 0.94 (0.88, 1.01) | 0.076 | 1.12 (0.86, 1.46) | 0.412 | 1.18 (0.76, 1.82) | 0.463 |  |  |
| Maternal illness in past month | 1.01 (0.95, 1.06) | 0.827 |  |  | 0.97 (0.86, 1.09) | 0.561 | 1.15 (0.95, 1.39) | 0.144 |  |  |
| Breast problems | 0.97 (0.86, 1.10) | 0.682 |  |  | 0.91 (0.63, 1.31) | 0.602 | 1.18 (0.92, 1.53) | 0.191 |  |  |
| Primary education and below (ref: above primary) | 0.96 (0.92, 1.01) | 0.108 |  |  | 0.96 (0.86, 1.07) | 0.466 | 1.11 (0.94, 1.32) | 0.203 |  |  |
| Married/cohabiting | 1.09 (0.97, 1.22) | 0.128 |  |  | 1.06 (0.92, 1.23) | 0.387 | 1.15 (0.92, 1.44) | 0.212 |  |  |
| (ref: other) |  |  |  |  |  |  |  |  |  |  |
| Mild, moderate or severe depression (ref: minimal or none) | 0.99 (0.93-1.04) | 0.594 |  |  | 0.79 (0.65, 0.97) | 0.024 | 1.00 (0.82, 1.23) | 0.963 |  |  |
| Intimate partner violence ≥10 HITS score (ref: <10) | 0.96 (0.86, 1.07) | 0.422 |  |  | 0.98 (0.85, 1.12) | 0.727 | 1.16 (0.99, 1.35) | 0.063 | 1.18 (0.98, 1.42) | 0.083 |
| Partner provides financial support (ref: no support) | 1.11 (0.98-1.26) | 0.096 |  |  | 1.19 (0.97, 1.45) | 0.095 | 1.04 (0.83, 1.31) | 0.716 |  |  |
| Low birth weight (ref: ≥2.5kg) | 0.98 (0.88, 1.09) | 0.727 |  |  | 0.95 (0.70, 1.30) | 0.767 | 0.50 (0.25, 0.99) | 0.048 | 0.50 (0.25, 0.99) | 0.046 |
| Prior infant hospitalization | 1.04 (1.01, 1.07) | 0.006 | 0.99 (0.96, 1.01) | 0.372 | 0.99 (0.81, 1.20) | 0.898 | 0.88 (0.64, 1.21) | 0.437 |  |  |
| Infant illness in past month | 0.99 (0.95, 1.04) | 0.781 |  |  | 0.99 (0.88, 1.10) | 0.809 | 1.19 (0.97, 1.44) | 0.088 | 1.19 (0.96, 1.48) | 0.107 |
| HIV disclose | 1.06 (0.97, 1.17) | 0.198 |  |  | 1.11 (0.89, 1.38) | 0.36 | 1.17 (0.84, 1.65) | 0.347 |  |  |
| On ART | 1.00 (0.96, 1.04) | 0.885 |  |  | 1.02 (0.91, 1.15) | 0.704 | 0.94 (0.78, 1.12) | 0.479 |  |  |

Abbreviations: aP, adjusted P value; aPR, adjusted prevalence ratio; ART, antiretroviral therapy; BMI, body mass index (kg/m^2^); CI, confidence interval; PR, prevalence ratio

^a^ Multivariable model included factors associated with breastfeeding practices at p<0.10; only mothers reporting ever breastfeeding were included in the analyses (10 women that did not initiate breastfeeding were excluded)

^b^ Exclusive breastfeeding at 6-week visit included only mothers at 6-week immunizations: age, mom BMI, have one child, and infant hospitalization

^c^ 6-monthly exclusive breastfeeding included only mothers at 9-month immunizations; was asked at the month-9 immunization visit: No multivariable model evaluated

^d^ Continued BF at 9-month visit included only mothers at 9-month immunizations: IPV, low birth weight and infant illness

Additional Table 3. Univariate and multivariable analysis of factors associated with breastfeeding practices among women not living with HIV ^a^

|  | | **Exclusive breastfeeding at 6-week visit^b^** | | | | **Exclusive breastfeeding during first 6-months^c^** | | | | **Continued breastfeeding at** | |
| --- | --- | --- | --- | --- | --- | --- | --- | --- | --- | --- | --- |
|  |  |  |  |  |  |  |  |  |  | **9-month visit^d^** | |
|  | **PR (95% CI)** | | **P** | **aPR (95% CI)** | **aP** | **PR (95% CI)** | **P** | **aPR (95% CI)** | **aP** | **PR (95% CI)** | **P** |
| Maternal age <25 years (ref: ≥25) | 0.97 (0.94, 1.00) | | 0.062 | 0.97 (0.94, 1.00) | 0.06 | 1.02 (0.94, 1.10) | 0.686 |  |  | 1.00 (0.99, 1.02) | 0.697 |
| Maternal BMI<18.5 (ref: ≥18.5) | 0.96 (0.87, 1.05) | | 0.351 |  |  | 1.01 (0.84, 1.21) | 0.918 |  |  | 0.99 (0.96, 1.04) | 0.806 |
| One child (ref: ≥2) | 1.01 (0.92, 1.09) | | 0.899 |  |  | 1.00 (0.80, 1.25) | 0.987 |  |  | 1.03 (0.96, 1.10) | 0.378 |
| Maternal illness in past month | 0.94 (0.89, 0.99) | | 0.016 | 0.95 (0.91, 1.00) | 0.072 | 0.98 (0.89, 1.08) | 0.644 |  |  | 1.00 (0.98, 1.02) | 0.866 |
| Breast problems | 0.95 (0.89, 1.02) | | 0.132 |  |  | 0.89 (0.76, 1.04) | 0.149 |  |  | 0.98 (0.95, 1.02) | 0.357 |
| Primary education and below (ref: above primary) | 0.95 (0.92, 0.98) | | 0.004 | 0.96 (0.93, 0.99) | 0.01 | 0.87 (0.79, 0.95) | 0.002 | 0.87 (0.79, 0.95) | 0.002 | 1.00 (0.98, 1.01) | 0.815 |
| Married/cohabiting (ref: other) | 1.01 (0.97, 1.05) | | 0.703 |  |  | 0.97 (0.85-1.10) | 0.58 |  |  | 1.01 (0.98, 1.04) | 0.402 |
| Mild, moderate or severe depression (ref: minimal or none) | 0.95 (0.88, 1.03) | | 0.241 |  |  | 0.86 (0.73, 1.01) | 0.073 | 0.93 (0.79, 1.10) | 0.411 | 0.98 (0.95, 1.02) | 0.351 |
| Intimate partner violence ≥10 HITS score (ref: <10) | 0.93 (0.86, 1.01) | | 0.069 | 0.95 (0.87, 1.04) | 0.282 | 0.75 (0.61, 0.91) | 0.004 | 0.78 (0.64, 0.95) | 0.015 | 0.98 (0.94, 1.02) | 0.277 |
| Partner provides financial support (ref: no support) | 1.01 (0.96, 1.07) | | 0.735 |  |  | 1.02 (0.88, 1.18) | 0.832 |  |  | 1.02 (0.98-1.06) | 0.276 |
| Low birth weight (ref: ≥2.5kg) | 0.91 (0.80, 1.02) | | 0.116 |  |  | 0.99 (0.81, 1.22) | 0.962 |  |  | 0.99 (0.94, 1.04) | 0.637 |
| Prior infant hospitalization | 0.96 (0.88, 1.06) | | 0.414 |  |  | 1.01 (0.88, 1.17) | 0.878 |  |  | 1.00 (0.97, 1.02) | 0.771 |
| Infant illness in past month | 0.95 (0.91, 0.99) | | 0.007 | 0.96 (0.93, 1.00) | 0.031 | 0.87 (0.79, 0.95) | 0.003 | 0.89 (0.80, 0.98) | 0.016 | 1.00 (0.98, 1.02) | 0.901 |

Abbreviations: aP, adjusted P value; aPR, adjusted prevalence ratio; BMI, body mass index (kg/m^2^); CI, confidence interval; PR, prevalence ratio

^a^ Multivariable model included factors associated with breastfeeding practices at p<0.10; ; only mothers reporting ever breastfeeding were included in the analyses (10 women that did not initiate breastfeeding were excluded)

^b^ Exclusive breastfeeding at 6-week visit included only mothers attending 6-week immunizations : maternal age, maternal illness, education status, IPV, and infant illness

^c^ 6-monthly exclusive breastfeeding included only mothers attending 9-month immunizations; was asked at the month-9 immunization visit: education status, maternal depression, IPV, and infant illness

^d^ Continued BF at 9-month visit included only mothers attending 9-month immunizations : No multivariable model evaluated
